# Desvendando um Enigma Genético: Hipertrofia Assimétrica em um Receptor de Transplante Cardíaco Relacionado à Síndrome de Birt-Hogg-Dubé

**DOI:** 10.36660/abc.20240444

**Published:** 2025-04-16

**Authors:** Silas Ramos Furquim, Lucas Vieira Lacerda Pires, Nara Alves Buriti, Mariana Lombardi Peres de Carvalho, Emanuelle Leonilia Marques, Bianca Domit Werner Linnenkamp, Luciana Sacilotto, Fabiana G. Marcondes-Braga, Iascara Wozniak de Campos, Luis Fernando Bernal da Costa Seguro, Sandrigo Mangini, Monica S. Avila, Fernando Bacal, José Eduardo Krieger

**Affiliations:** 1 Hospital das Clínicas Faculdade de Medicina Universidade de São Paulo Paulo SP Brasil Instituto do Coração do Hospital das Clínicas da Faculdade de Medicina da Universidade de São Paulo, São Paulo, SP – Brasil

**Keywords:** Cardiomiopatia Hipertrófica, Transplante de Coração, Genética

## Abstract

Um caso de um homem de 54 anos que se submeteu a um transplante cardíaco com hipertrofia assimétrica precoce não relacionada à rejeição. A análise genética do doador revelou uma variante no gene da foliculina, associada à síndrome de Birt-Hogg-Dubé. O rastreamento da família do doador identificou uma hipertrofia similar e a mesma variante genética no pai do doador. Embora a avaliação genética dos tecidos dos doadores não seja realizada rotineiramente, ela pode ser crucial para compreender alterações não relacionadas ao procedimento do transplante e identificar carreadores de variantes patogênicas.

## Introdução

O aumento na espessura da parede ventricular esquerda, não explicado por condições de sobrecarga, é conhecido como cardiomiopatia hipertrófica. Essa condição é primariamente causada por variantes nos genes sarcoméricos, embora 5-10% dos casos possam envolver outros fatores genéticos.^1^O desenvolvimento de hipertrofia ventricular esquerda (HVE) pós-transplante não é incomum, e as causas incluem hipertensão induzida por inibidores de calcineurina, efeitos de imunossupressão e lesão imune.^2^ Este relato destaca um caso atípico de HVE que ocorreu imediatamente após o transplante, relacionada a uma variante genética específica. A descoberta da variante facilitou o rastreamento familiar.

## Relato de Caso

**Figura 1:**
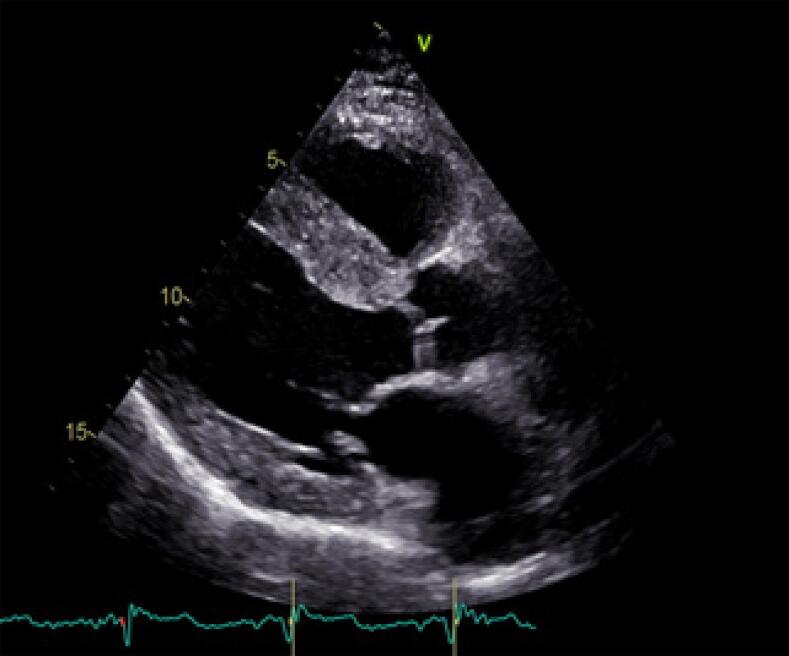
– Ecocardiograma após o transplante cardíaco.

**Figura 2:**
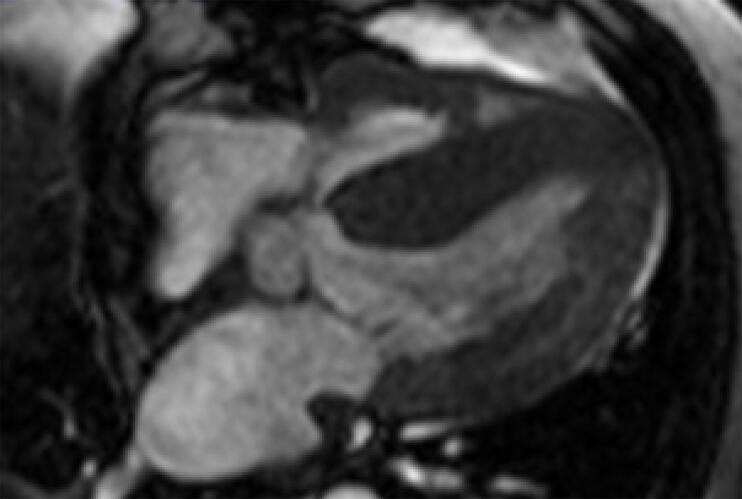
– Ressonância magnética cardíaca após o transplante.

Um homem de 54 anos, com cardiomiopatia dilatada avançada, recebeu um transplante cardíaco (TC) de um doador de 44 anos que foi a óbito por hemorragia subaracnóidea. O histórico clínico do doador incluia hipertensão e tabagismo. Antes do TC, foi realizado um ecocardiograma à beira-leito no hospital do doador, que não indicou anormalidades estruturais importantes. O TC foi realizado sem complicações, e o paciente passou por um processo padrão de recuperação. No entanto, o ecocardiograma pós-operatório revelou hipertrofia assimétrica no septo interventricular medindo 16mm, e fração de ejeção ventricular esquerda normal (Figura 1). Essa hipertrofia foi confirmada por ressonância magnética cardíaca, que mostrou uma espessura máxima de 20mm na região média anterosseptal, sem obstrução da via de saída (Figura 2). Rejeição foi descartada após duas biópsias endomiocárdicas realizadas com intervalo de oito dias, que mostraram resultados 1R e PAMR0.

### Seguimento

A pressão arterial do paciente foi controlada com enalapril (15mg/ dia) e diltiazem (180 mg/dia). Contudo, os ecocardiogramas mostraram consistentemente hipertrofia septal assimétrica. O paciente foi submetido a três biópsias adicionais, sem sinais de rejeição celular ou humoral. O painel imunológico foi negativo para antígenos classe I e II, em um regime de ciclosporina, micofenolato e prednisona.

Assim, consideramos uma doença genética relacionada aos genes sarcoméricos no doador, apesar do ecocardiograma prévio normal. O sequenciamento de exoma completo foi conduzido no DNA de células esplênicas armazenadas do doador, inicialmente coletadas para teste de histocompatibilidade.

A análise revelou uma variante *frameshift* em heterozigose no gene da foliculina - *FLCN* (NM_144997.7):c.1285dupC:p.(His429Profs*27). Essa variante foi classificada como patogênica de acordo com as diretrizes do *American College of Medical Genetics* e do *Genomics/Association for Molecular Pathology*.

### Rastreamento familiar

**Figura 3:**
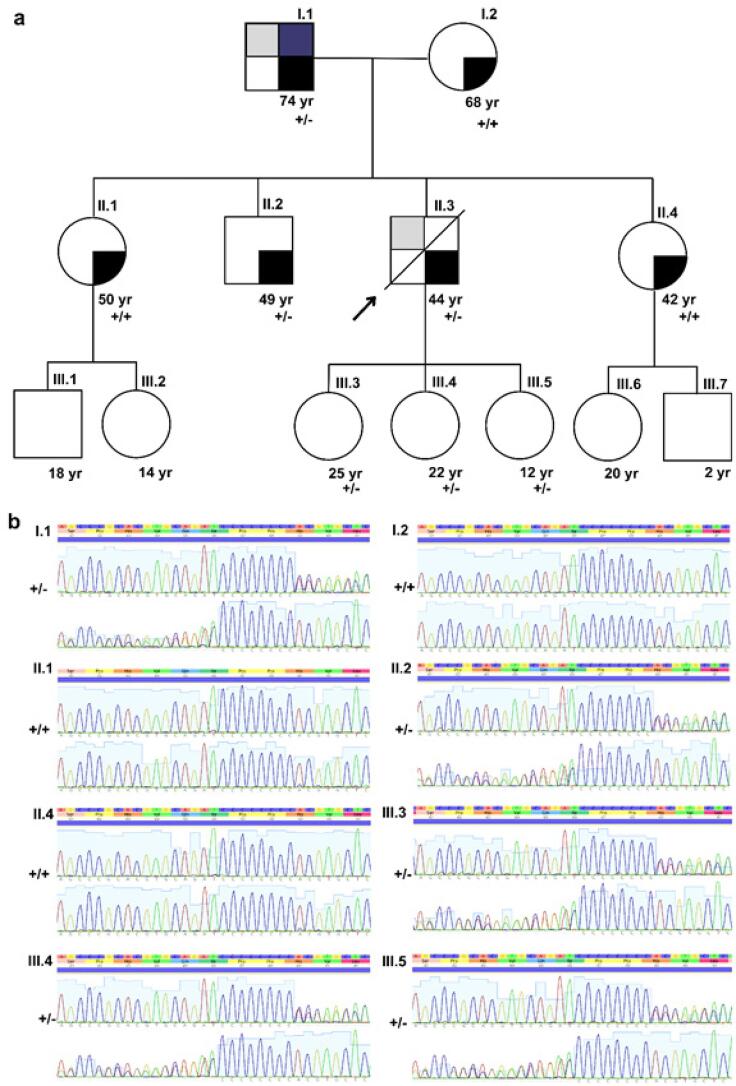
– Pedigree familiar e achados genéticos da variante do gene da foliculina (FLCN) (NM_144997.7):c.1285dupC:p.(His429Profs*27). A) pedigree da família do doador; quadrados e círculos indicam homens e mulheres, respectivamente; a seta indica o paciente índice (doador do coração); linhas diagonais indicam indivíduos falecidos. As cores representam os fenótipos dos familiares: cinza para hipertrofia, azul para múltiplos fibrofoliculomas cutâneos, e preto para hipertensão. Alelo do tipo selvagem está representado por “+” e alelo do gene da FLCN p.His429Profs*27 está representado por “-“ (+/+ para indivíduos selvagens e +/- para heterozigotos). B) Cromatogramas gerados por sequenciamento de Sanger dos familiares rastreou a presença da variação do gene da FLCN p.His429Profs*27 (+/+ para indivíduos selvagens e +/- para heterozigotos).

Após a identificação da variante hereditária, nós informamos a central estadual de transplante para alertar a família do doador sobre sua presença. Oferecemos avaliações cardiológicas e genéticas aos familiares, que compareceram às avaliações clínicas. Realizado heredograma (Figura 3), exame clínico, eletrocardiograma, ecocardiograma e o sequenciamento (Sanger) para detectar a variante nos familiares.

**Figura 4:**
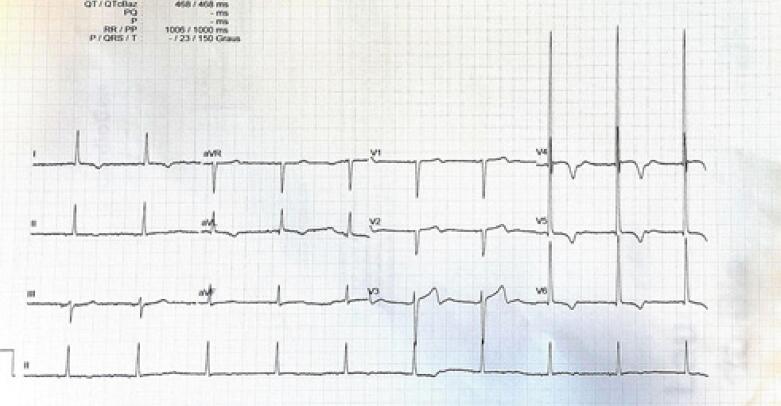
– Eletrocardiograma do pai do doador sugestivo de hipertrofia ventricular esquerda.

As avaliações revelaram a mesma hipertrofia septal assimétrica (17 mm) e a idêntica variante genética no pai do doador (indivíduo I.1), conforme ilustrado nas Figuras 4 e 5. Clinicamente, o pai do doador apresentava múltiplos fibrofoliculomas cutâneos, um importante critério diagnóstico para Síndrome de Birt-Hogg-Dubé (SBHD), em conformidade com achados moleculares. Como a SBHD pode aumentar o risco de vários tumores, o pai do doador foi orientado a se submeter a um rastreamento oncológico abrangente.

**Figura 5:**
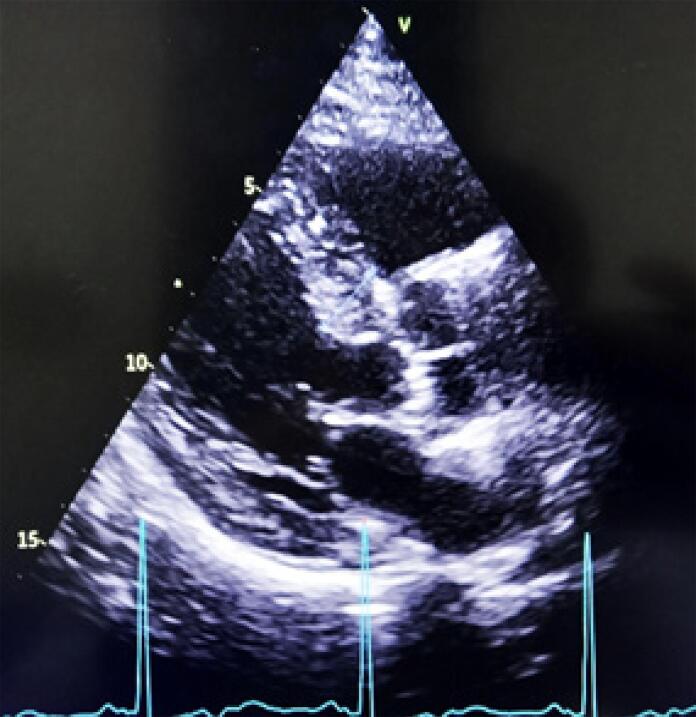
– Ecocardiograma transtorácico do pai do doador mostrando septo anterior basal com 17 mm.

Quanto aos irmãos do doador, observou-se que seu irmão carregava a mesma variante genética, embora sem hipertrofia cardíaca, enquanto suas irmãs não possuíam a variante nem foliculomas cutâneos. Consequentemente, o irmão do doador foi encaminhado para um rastreamento mais amplo das principais manifestações clínicas da SBHD.

## Discussão

A HVE é uma condição comum pós-TC, que afeta até 83% dos pacientes em um ano, aumentando o risco de morte em 1,9 vezes.^2^ As causas da HVE incluem hipertensão causada por inibidores de calcineurina, imunossupressão, e lesão imune.^2^ A apresentação precoce não usual de hipertrofia assimétrica após o transplante gerou preocupações no processo de doação do órgão.

Pesquisas recentes sugerem que a HVE em aloenxertos pode estar associada a vias de sinalização pró-fibrótica e ativação da proteína alvo da rapamicina em mamíferos (mTOR), sugerindo potenciais implicações terapêuticas.^3^ O estudo RADTAC revelou que uma combinação de everolimus e tacrolimus em baixa dose reduz a HVE em comparação ao tacrolimus em dose padrão em um ano após o TC.^4^

Cardiomiócitos hipertrofiados exibem síntese proteica aumentada e estímulo de crescimento, principalmente pela via de sinalização AMPK-mTOR. O gene da *FLCN*, associado a essa via, codifica uma proteína envolvida em várias sinalizações. A perda da função da *FLCN* tem sido relacionada à desregulação da via mTOR. Ainda, variantes patogênicas no gene da proteína interativa com foliculina 1 (*FNIP1*) foram associadas à déficit imunológico e hipertrofia cardíaca. Modelos animais sugerem que o *knockout* do gene da *FLCN* leva à hipertrofia cardíaca, modulada pela via mTOR.^5,6^

Variantes patogênicas em ambos os alelos da *FNIP1* foram associadas a uma condição caracterizada por disfunção imune e hipertrofia cardíaca.^7-9^ Estudos usando modelos animais revelaram que a deleção do gene da *FLNC* leva a um aumento do coração em camundongos, acompanhado de uma redução na fosforilação de AMPKα e atividade da mTORC1 aumentada. O tratamento subsequente com um inibidor da via mTOR reverteu o aumento cardíaco e melhorou marcantemente a função ventricular. Tal fato destaca a conexão previamente estabelecida entre desregulação do mTOR e haploinsuficiência da *FLCN* e sugere essa via específica como um potencial alvo terapêutico.^10^ Variantes no gene da *FLCN* tipicamente resultam em SBHD, caracterizada por neoplasias renais, fibrofoliculomas, e cistos pulmonares.^11^ Esse caso pode representar a primeira relação conhecida entre hipertrofia cardíaca e SBHD.

A avaliação genética dos tecidos do doador, apesar de não ser padrão, foi crucial neste caso. O rastreamento familiar após a identificação da variante é vital para o seguimento clínico e rastreamento para câncer entre os que carregam a variante patogênica. Neste caso, a cossegregação da variante na família do doador apoia e desafia a associação com a HVE, destacando a necessidade de mais pesquisas.

## Conclusões

A HVE não é tipicamente vista precocemente após o transplante, e geralmente está associada a variantes genéticas sarcoméricas. Este relato é provavelmente o primeiro a associar a *FLCN* com hipertrofia assimétrica, o que foi corroborado por rastreamento familiar. Isso destaca a importância e a viabilidade da avaliação genética dos tecidos do doador, ampliando o entendimento das complicações pós-transplante e melhorando o cuidado do paciente.
