# Supplementary material for: Desvendando um Enigma Genético: Hipertrofia Assimétrica em um Receptor de Transplante Cardíaco Relacionado à Síndrome de Birt-Hogg-Dubé
Source: Arq Bras Cardiol. 2025 Apr 16;122(3):e20240444. [Article in Portuguese] doi: 10.36660/abc.20240444 (PMC12133066; doi:10.36660/abc.20240444)
Supplement: Translation

# Translation: Unveiling the Genetic Puzzle: Asymmetric Hypertrophy in a Heart Transplant Recipient Linked to Birt-Hogg-Dubé Syndrome

**DOI:** 10.36660/abc.20240444i

**Authors:** Silas Ramos Furquim, Lucas Vieira Lacerda Pires, Nara Alves Buriti, Mariana Lombardi Peres de Carvalho, Emanuelle Leonilia Marques, Bianca Domit Werner Linnenkamp, Luciana Sacilotto, Fabiana G. Marcondes-Braga, Iascara Wozniak de Campos, Luis Fernando Bernal da Costa Seguro, Sandrigo Mangini, Monica S. Avila, Fernando Bacal, José Eduardo Krieger

**Affiliations:** 1 Brazil Instituto do Coração do Hospital das Clínicas da Faculdade de Medicina da Universidade de São Paulo, São Paulo, SP – Brazil

**Keywords:** Hypertrophic Cardiomyopathy, Heart Transplantation, Genetics

## Abstract

A case of a 54-year-old man who underwent a heart transplant with early asymmetric hypertrophy unrelated to rejection. Donor’s genetic analysis revealed a variant in folliculin gene, associated with Birt-Hogg-Dubé Syndrome. Screening of the donor’s family uncovered a similar hypertrophy and same genetic variant in the donor’s father. Although genetic evaluation of donors’ tissues is not routine, it can be crucial for understanding changes unrelated to the transplant procedure and identifying carriers of pathogenic variants.

## Introduction

Increased left ventricle (LV) wall thickness non explained by loading conditions is known as hypertrophic cardiomyopathy. This condition is primarily caused by genetic variants in sarcomeric genes, though 5-10% of cases may involve other genetic factors.^1^ The development of post-transplantation LV hypertrophy (LVH) is not infrequent, with causes including hypertension induced by calcineurin inhibitors, effects of immunosuppression, and immune injury.^2^ This report highlights an atypical case of LVH occurring immediately after transplantation, linked to a specific genetic variant. The variant discovery facilitated family screening.

## Case Report

A 54-year-old male, with advanced dilated cardiomyopathy, received a heart transplant (HT) from a 44-year-old donor who had succumbed to an arachnoid hemorrhage. The donor’s medical background included hypertension and tobacco usage. Prior to HT, evaluations included a bedside echocardiogram conducted at the donor’s hospital, which indicated no gross structural abnormalities. The HT procedure was performed without complications, and the patient experienced a standard recovery process. However, a postoperative echocardiogram revealed asymmetric hypertrophy in the interventricular septum measuring 16 mm, and normal LV ejection fraction (Figure 1). This hypertrophy was further confirmed by cardiac magnetic resonance imaging, which showed a maximum thickness of 20 mm in the mid-anteroseptal region, without systolic obstruction (Figure 2). Rejection was ruled out following two endomyocardial biopsies conducted eight days apart, which showed 1R and PAMR0 results.

**Figure 1:** – Echocardiogram after cardiac transplantation.

**Figure 2:** – Cardiac magnetic resonance after cardiac transplantation.

### Follow-up

Patient’s blood pressure was well-managed with enalapril (15 mg/day) and diltiazem (180 mg/day). However, echocardiograms consistently showed asymmetrical septal hypertrophy. The patient underwent three additional biopsies, with no signs of humoral or cellular rejection. An immunological panel also returned negative for class I and II antigens, on a regimen of cyclosporine, mycophenolate, and prednisone.

Hence, we considered a genetic disease related to sarcomeric genes in the heart donor despite the previously reported normal echocardiogram. Whole exome sequencing was conducted on DNA from the donor’s stored splenic cells, initially collected for histocompatibility testing.

Remarkably, analysis uncovered a heterozygous frameshift variant in folliculin gene - *FLCN* (NM_144997.7):c.1285dupC:p.(His429Profs*27). This variant was classified as pathogenic according to the American College of Medical Genetics and Genomics/Association for Molecular Pathology guidelines.

### Family screening

Following hereditary variant identification, we informed the state transplantation center to alert the donor’s family about its presence. We offered cardiological and genetic assessments to family members, who then attended clinical evaluations. We constructed a family pedigree (Figure 3), conducted comprehensive clinical examinations, ECGs, echocardiograms, and Sanger sequencing to detect the variant in relatives.

**Figure 3:** – Family pedigree and genetic findings of folliculin (FLCN) gene variant (NM_144997.7):c.1285dupC:p.(His429Profs*27). A) Donor’s family pedigree; squares and circles indicate males and females respectively; the arrow indicates index patient (heart donor). Diagonal lines indicate deceased individuals. Colors represent family member’s phenotypes; gray for cardiac hypertrophy, blue for multiple cutaneous fibrofolliculomas and black hypertension. Wildtype allele is represented by “+” and FLCN p.His429Profs*27 allele is represented by “-“ (+/+ for wildtype individuals and +/- for heterozygotes). B) Chromatograms generated by Sanger sequencing of family members screened for FLCN p.His429Profs*27 variation. (+/+ for wildtype individuals and +/- for heterozygotes).

These assessments revealed the same asymmetric septal hypertrophy (17 mm) and the identical genetic variant in the donor’s father (individual I.1), as shown in Figures 4 and 5. Clinically, donor’s father exhibited multiple cutaneous fibrofolliculomas, a major diagnostic criterion for Birt-Hogg-Dubé syndrome (BHDS), aligning with molecular findings. Given that BHDS can increase the risk of various tumors, the donor’s father was advised to undergo comprehensive cancer screening.

**Figure 4:** – Donor´s father electrocardiogram suggestive of left ventricular hypertrophy.

**Figure 5:** – Donor´s father transthoracic echocardiogram showing basal anterior septum with 17 mm.

Regarding donor’s siblings, his brother was found to carry the same genetic variant, though without any cardiac hypertrophy, while his sisters did not have the variant and no visible cutaneous folliculomas. Consequently, the donor’s brother was referred for broader screening to cover the primary clinical manifestations of BHDS.

## Discussion

LVH is a common post-HT condition, affecting up to 83% of patients within a year and increasing mortality risk by 1.9 times.^2^ Causes of LVH include hypertension due to calcineurin inhibitors, immunosuppression, and immune injury.^2^ Unusual early presentation of asymmetric hypertrophy post-transplant could raise concerns in the organ donation process.

Emerging research suggests that LVH in allografts may be linked to pro-fibrotic signaling pathways and mammalian target of rapamycin (mTOR) activation, suggesting potential therapeutic implications.^3^ The RADTAC study revealed that a combination of low-dose everolimus and tacrolimus reduces LVH compared to standard-dose tacrolimus one year post-HT.^4^

Hypertrophied cardiomyocytes exhibit increased protein synthesis and growth stimulus, primarily through the AMPK-mTOR signaling pathway. *FLCN* gene, associated with this pathway, encodes a protein implicated in various intracellular signals. Loss of *FLCN* function has been tied to mTOR pathway deregulation. Moreover, pathogenic variants in Folliculin-interacting protein 1 (*FNIP1*) have been linked to immunological impairment and cardiac hypertrophy. Animal models suggest that *FLCN* knockout leads to heart hypertrophy, modulated by the mTOR pathway.^5,6^ Pathogenic variants in both alleles of *FNIP1* have been linked to a condition characterized by immune dysfunction and cardiac hypertrophy.^7-9^ Studies using animal models revealed that *FLNC* gene deletion leads to heart enlargement in mice accompanied by reduced phosphorylation of AMPKα and heightened mTORC1 activity. Subsequent treatment with an inhibitor of the mTOR pathway reversed the cardiac enlargement and markedly improved heart function. This underscores the previously established connection between mTOR dysregulation and *FLCN* haploinsufficiency and suggests targeting this pathway as a potential therapeutic approach.^10^ *FLCN* gene mutations typically result in BHDS, characterized by renal neoplasms, fibrofolliculomas, and lung cysts.^11^ This case may represent the first known link between cardiac hypertrophy and BHDS.

Genetic evaluation of donor’s tissues, although not standard, was crucial in this case. Family screening following variant identification is vital for clinical follow-up and cancer screening for those carrying the pathogenic variant. In this case, variant cosegregation within the donor’s family both supports and challenges LVH association, highlighting the need for further research.

## Conclusions

LVH is not typically seen early after transplantation, and asymmetric hypertrophy is often linked to sarcomeric gene variants. This report is possibly the first to associate *FLCN* with asymmetrical hypertrophy, supported by familial screening. It underscores the importance and feasibility of genetic evaluation of donor’s tissues, broadening the understanding of post-transplant complications and enhancing patient care.
